# Left Ventricular Thrombus in a 34-year-old Female Seen on Point-of-care Ultrasound

**DOI:** 10.5811/cpcem.2019.1.40801

**Published:** 2019-01-22

**Authors:** Faraz Khan, Shadi Lahham

**Affiliations:** * University of California, Irvine School of Medicine, Irvine, California; † University of California, Irvine, Department of Emergency Medicine, Orange, California

## CASE PRESENTATION

A 34-year-old female with a history of methamphetamine-associated cardiomyopathy presented to the emergency department (ED) with generalized weakness, altered mental status, and chest pain. She reported a recent placement of an automatic implantable cardioverter-defibrillator at an outside hospital three months prior to current presentation and had a documented ejection fraction of 15%. Upon arrival to the ED, she was hypotensive with a systolic blood pressure ranging in the 40s to 70s millimeters of mercury and was hypothermic at 33.6 degrees Celsius. She appeared cachectic and had a 3/6 systolic ejection murmur at the left upper sternal border. We performed a point-of-care ultrasound (POCUS) to assess the patient’s cardiac function and found a large left ventricular (LV) thrombus measuring 5.8 × 2.8 centimeters (Image). Further views of the thrombus seen in the video reveal a large hyperechoic density in the left ventricle. The patient was admitted to the intensive care unit for vasopressor support and thrombolytic therapy.

## DISCUSSION

Cardiovascular disease is the leading cause of death in patients with methamphetamine use, and cardiomyopathy is a rare complication that can occur.^1^ This can lead to systolic dysfunction and reduced ejection fraction, which is an important risk factor for the formation of LV thrombi.^2^ In patients with methamphetamine-associated cardiomyopathy with an ejection fraction less than 40%, up to 33% can develop a LV thrombus.^3^ POCUS can be used to help diagnose patients with an LV thrombus.^4^ Patients found to have a thrombus should be started on anticoagulation therapy.^5^

**CPC-EM Capsule**

What do we already know about this clinical entity?

*Left ventricular thrombus is a complication of cardiomyopathy and can present with shortness of breath and fatigue*.

What is the major impact of the image(s)?

*These images depict a left ventricular thrombus as seen on point-of-care ultrasound (POCUS)*.

How might this improve emergency medicine practice?

*Emergency physicians can use POCUS to quickly identify a left ventricular thrombus*.

## Supplementary Information

Video A hyperechoic mobile structure within the left ventricle is seen using point-of-care ultrasound through the parasternal long axis, parasternal short axis, and apical four-chamber views of the heart.

## Figures and Tables

**Image:**
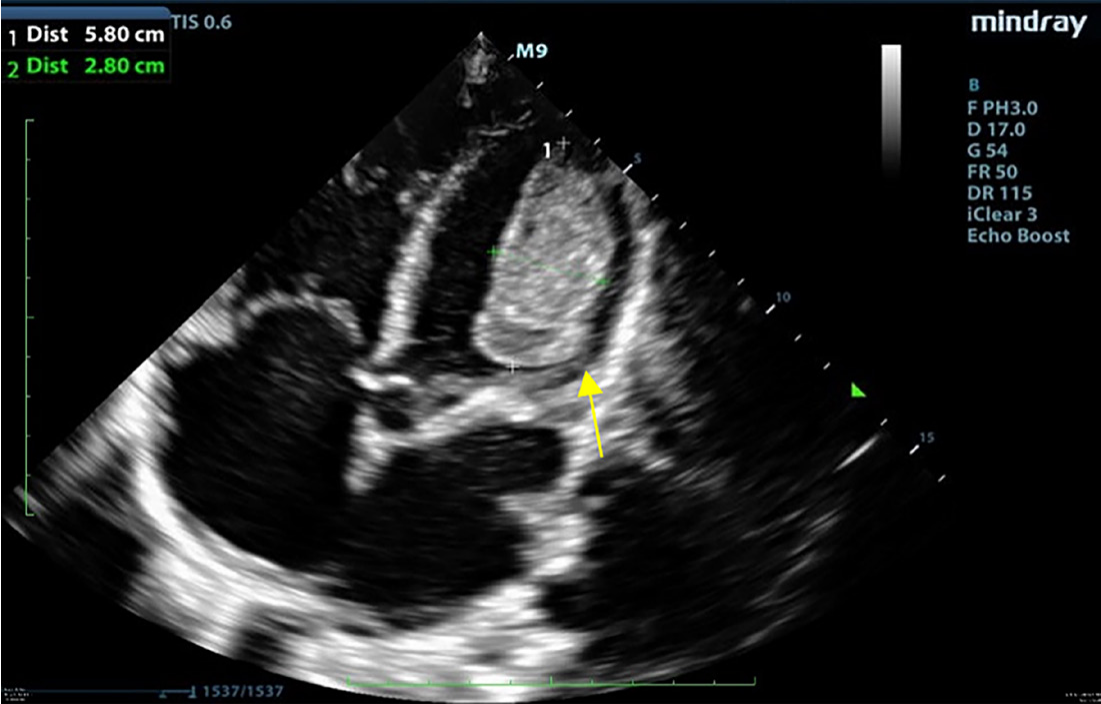
Apical four-chamber view of the heart showing a thrombus in the left ventricle measuring 5.8 × 2.8 centimeters (arrow).
